# Research training experience of Vietnamese health sciences undergraduates visiting the Centre for the AIDS Programme of Research in South Africa

**DOI:** 10.11604/pamj.2023.44.109.39039

**Published:** 2023-02-27

**Authors:** Andrew William Taylor-Robinson, Lenine Liebenberg

**Affiliations:** 1 College of Health Sciences, VinUniversity, Gia Lam District, Hanoi, Vietnam,; 2 Center for Global Health, Perelman School of Medicine, University of Pennsylvania, Philadelphia, USA,; 3 Centre for the AIDS Programme of Research in South Africa (CAPRISA), Durban, South Africa,; 4 Department of Medical Microbiology, University of KwaZulu-Natal, Mayville, South Africa

**Keywords:** Research, training, mentorship programme, health sciences education, HIV, tuberculosis

## Abstract

The Centre for the AIDS Programme of Research in South Africa (CAPRISA), performs world-leading research on the epidemiology, pathogenesis, prevention and treatment of HIV/AIDS, tuberculosis, and - more recently - COVID-19. A rigorous yet supportive academic culture has nurtured the careers of many successful health sciences researchers, some of whom have worked for the organization since its inception over 20 years ago. This focus on professional development is founded on a training programme that invests heavily in the individual with the payoff of strengthening the science base for HIV and tuberculosis research in South Africa. Those selected for mentorship are typically medical students from the University of KwaZulu-Natal, adjoining the headquarters of CAPRISA in Durban. Increasingly, however, the institute attracts international fellows from partnering organizations to experience the intellectually demanding, scientifically robust, cutting-edge research environment. The purpose of this voices piece is to narrate and critically evaluate the experience from the dual perspectives of host and visitor of a research training programme undertaken by three undergraduate health sciences students from Vietnam, enrolled at VinUniversity. This was the inaugural running of what is expected to be an annual summer trip to CAPRISA by Hanoi-based medical and nursing students. The formative educational experience in best practice tackling of infectious diseases in challenging clinical contexts demonstrated the importance of investing in research placement programmes for public health impact. The exchange has inspired each student to become a future leader in seeking bold, innovative, and strategic approaches to improve global health issues in their home country.

## Perspective

This critical reflection describes the experiential learning of Vietnamese undergraduates conducting a two-week intensive research training visit to South Africa. The one nursing and two medical students came from the College of Health Sciences, VinUniversity, in Hanoi. Although operating for just two years this start-up university has already attained an overall 4-star Quacquarelli Symonds (QS) ranking, with 5-star teaching quality, the fastest tertiary education institution in the Asia-Pacific region to reach this educator provider milestone [1]. This has been achieved primarily through ongoing strategic partnerships with two of the world´s leading universities, Cornell and the University of Pennsylvania. These valuable relationships are helping VinUniversity to establish its faculty and leadership teams, to validate and assure the quality of the curriculum, to guide international accreditation, and offer students opportunities to intern and study with academic partners and collaborators in Vietnam and abroad.

It is the last of these facets that is highlighted by the nascent partnership between VinUniversity and CAPRISA, the Centre for the AIDS Programme of Research in South Africa. In January 2022, Professors Salim and Quarraisha Abdool Karim, the Director and Associate Scientific Director, were jointly awarded the inaugural VinFuture Special Prize for Innovators from Developing Countries in recognition of CAPRISA's ground-breaking research on prevention and treatment of infection with human immunodeficiency virus (HIV), the cause of acquired immunodeficiency syndrome AIDS [2]. Further to this, the two organizations signed a memorandum of understanding for CAPRISA to annually host selected healthcare profession students from VinUniversity during the northern hemisphere summer vacation prior to the start of their clinical studies. During August 2022, the Durban headquarters of CAPRISA, based on the University of KwaZulu-Natal (UKZN) medical school campus, welcomed the first cohort of visitors, which included the corresponding author as accompanying faculty member, to engage in a coordinated schedule of research training and mentoring activities [3].

Each selected student was a strong academic performer with a proven research interest in public health, community medicine, epidemiology and/or infectious diseases. They also demonstrated curiosity, collaboration, organization, conscientiousness, resilience and adaptability, all valued attributes of medical researchers in a clinical setting. The rigorous selection process involved the candidate´s submission of a written application including resume and a letter of interest in which they outlined their qualifications, experience and motivation. From over 30 applications, six students were shortlisted for a 30-minute interview. The four-member panel comprised the Dean of the College of Health Sciences, the Director of each of the Medicine and Nursing programs, plus the corresponding author as relevant discipline-specific faculty. The six selection criteria were: academic performance; research experience (preferably of direct relevance); subject-specific interest and knowledge; commitment and motivation; English proficiency (verbal and written); social skills, collegiality and ambassadorship. Each was scored by all panel members on a scale of 1-5 with regard to expectations: i) not met; ii) partially met; iii) met; iv) partially exceeded; v) exceeded (overall score out of 30 x 4 = 120). Interviewed candidates were also asked to confirm their: availability for the planned travel dates (with scope for possible change of itinerary); possession of a valid passport with a minimum of six months to expiry, with visa to be arranged; possession of an authorized COVID-19 booster vaccination certificate.

The primary aim of the trip was for the VinUniversity students to gain insights into CAPRISA's world class research in prevention, control and treatment of HIV and tuberculosis (TB), the most common opportunistic infection in patients, in urban and rural settings. South Africa remains at the epicentre of the HIV pandemic, with the world's highest number of new infections but also the largest treatment programme [4]. Tuberculosis is the leading cause of death among HIV-infected individuals in the country. CAPRISA conducts research in four interrelated scientific programs, namely: HIV and TB treatment; microbicides; prevention and epidemiology; pathogenesis and vaccines. The enriching activities included visits to CAPRISA´s clinical research sites and meetings with the leadership, scientists, clinicians, staff and students. The three clinics attended varied in research focus, patient recruitment and community location. These were: Ethekwini, which adjoins the Prince Cyril Zulu Communicable Disease Centre, the largest government out-patient TB and sexually transmitted diseases treatment facility in Durban city centre; Springfield, attached to the King Dinuzulu Hospital, a large provincial TB referral hospital within a Durban suburb; and Vulindlela, next to the Mafakatini primary health care clinic in the rural Vulindlela district of KwaZulu-Natal (KZN), 120 km northeast of Durban. A notable highlight was the opportunity to participate in impactful community outreach programmes, such as civic leaders´ engagement workshops organized by the KZN Provincial Council on AIDS and by the National Government of South Africa Department of Women, Youth and Persons with Disabilities.

As research conducted by CAPRISA is not only locally responsive but also globally relevant, this initiated lively discussion on how to translate the outcomes of studies in KZN to Vietnam where cultural norms and social constructs are very different. There is a marked distinction between the sociodemographic groups of greatest vulnerability in the two settings. In South Africa, prevalence of HIV is highest in the heterosexual community, notably adolescent and young adult females in transactional relationships [4]; in Vietnam, infection foci are concentrated among men who have sex with men and people who inject drugs [5]. Furthermore, in South Africa, rural and peri-urban areas remain a focus of HIV infection. In contrast, Vietnam maintains a predominantly metropolitan public health concern, particularly in Hanoi and Ho Chi Minh City (formerly Saigon), Vietnam´s two largest cities and the twin hubs of this fast-developing Southeast Asian country.

Regardless of nation, for transmission of HIV undetectable equals untransmissible, U = U (or K = K, in Vietnamese) [6]. While acknowledging contrasting epidemiological trends and risk factors that are associated with different sexual practices in the two countries [4,5], common themes were recognized regarding outreach activities to engage, inform and change behaviours of susceptible individuals. In all contexts, protected sex - ostensibly, the use of condoms - is to be encouraged. This requires understanding customs and conventions in order to sensitively address entrenched attitudes, stigmas and taboos. U = U is incredibly powerful as an HIV public health slogan. Even more importantly, the message and the science behind it are life-changing for people living with HIV (PLHIV), their partners and families.

With the advent of highly effective HIV biomedical treatment and prevention, persons living with HIV enjoy longer and much healthier lives than experienced by their counterparts in previous generations whose condition, without effective antiviral therapy, might have progressed to full-blown AIDS. Moreover, persons at risk for HIV have more options than they did for staying HIV negative. Nevertheless, those affected by HIV continue to face stigma and discrimination, hindering access to care. This appears to be more of an issue in Vietnam, where social and sexual mores are traditionally conservative, especially in the north of the country. Against this backdrop, healthcare workers play crucial roles in striving to create an equitable, supportive and friendly environment for key populations, particularly among the more sexually experimental and less easily affronted younger generations, thereby enabling Vietnam to meet ambitious HIV epidemic control targets by 2030 [7].

From the post-trip testimonies of the three students this proved a transformative educational experience for them to witness best practice in HIV/AIDS awareness, treatment and prevention in KZN. It is from their pioneering cohort that VinUniversity envisages that tomorrow´s leaders of the healthcare professions will emerge to drive critically needed improvements to the public healthcare system in Vietnam, a nation with a median age of 32 years and whose population will pass 100 million in 2023. The CAPRISA ethos of excellence, be that in academic studies, clinical practice or research endeavours, is now instilled in each student. This philosophy compliments the aspirational graduate attributes of all VinUnians, the qualities of Excel: empathy; exceptional ability; creativity; entrepreneurial mindset; and leadership spirit.

Peer-assisted learning is a broadly practised learner-led educational model in health sciences curricula. This aims to limit the negative power dynamics that may be even subtly experienced by undergraduates and foundation-year trainee doctors and nurses when interacting with a senior teacher, typically a professor or consultant (often unbeknown to that lecturer/supervisor/tutor/ mentor) [8]. One particularly valuable aspect of the trip was the sharing of experiences with CAPRISA's own cohort of mentored clinical research placement fellows. These were participating undergraduate students from Nelson R. Mandela School of Medicine and other schools at University of UKZN, as well as postgraduate researchers at various stages of project progression. It was soon realized that regardless of sociocultural context, students from both South Africa and Vietnam had a shared purpose and experienced common problems upon which they felt able to reflect in this relatively safe environment. Through intensive peer support discussions, and by meeting with the co-author who is a prominent advocate of reflective practice in health professions education and the role of mentoring to develop skills and expertise in a research environment, each VinUniversity student was able to identify a personalized strategy to help overcome common challenges associated with biomedical research training.

Broadened perspectives and experiences through international, interinstitutional, multi-disciplinary research placement programmes have the potential to develop leaders that generate innovative solutions to public health concerns. This research experience visit to CAPRISA was the first external outreach and engagement activity for the inaugural intake of VinUniversity health sciences students. Its critically acclaimed success from both stakeholders has secured the collaboration between the two organizations. Discussions are well advanced to make the annual exchange bidirectional, enabling CAPRISA-mentored UKZN students a reciprocal opportunity to visit Hanoi. This would involve public health research and community engagement activities at VinUniversity College of Health Sciences and its partner healthcare providers, principally the Joint Commission International-accredited Vinmec Times City International Hospital. Selected students would also experience the impactful work of the United States Centers for Disease Control and Prevention, which provide technical expertise to the Vietnam Ministry of Health to support programmes that target HIV and TB as priority infectious diseases.
